# Massive Congestive Facial and Submandibular Oedema Due to Extreme Neck Flexion Following Suboccipital Craniectomy: A Case Report

**DOI:** 10.7759/cureus.31759

**Published:** 2022-11-21

**Authors:** Balaji Vaithialingam, Dheeraj Masapu, Satish Rudrappa

**Affiliations:** 1 Department of Anaesthesiology, Sakra World Hospital, Bengaluru, IND; 2 Department of Neurosciences, Sakra World Hospital, Bengaluru, IND

**Keywords:** neck flexion, submandibular swelling, suboccipital craniectomy, arnold chiari malformation, congestive oedema

## Abstract

A variety of factors could contribute to facial oedema during a prone neurosurgical procedure. For optimal surgical exposure, suboccipital cranial surgeries frequently necessitate extreme neck flexion. Extreme neck flexion in the prone position can impair venous drainage of the facial and oropharyngeal structures, leading to life-threatening oedema, so a two-fingerbreadth space between the chin and the sternum is critical. We present a case of massive facial oedema with submandibular swelling in a patient who underwent foramen magnum decompression in the prone position for Arnold Chiari malformation.

## Introduction

Suboccipital cranial surgeries are commonly used to treat pathologies of the posterior cranial fossa and Arnold Chiari malformation (ACM) [1]. The standard operative procedure for patients with ACM is foramen magnum decompression with C1 arch excision. In this patient population, good neck flexion in the prone position is required for a successful posterior fossa decompression. Prolonged surgeries in the prone can result in oropharyngeal swelling, postoperative vision loss, abdominal and limb compartment syndromes, pressure sores, and nerve palsies [2]. We report a case of severe congestive facial oedema with submandibular swelling due to extreme neck flexion in the prone position after suboccipital craniectomy in a patient with ACM. The patient and his family provided written and informed consent for the publication of this case report.

## Case presentation

**Figure 1:**
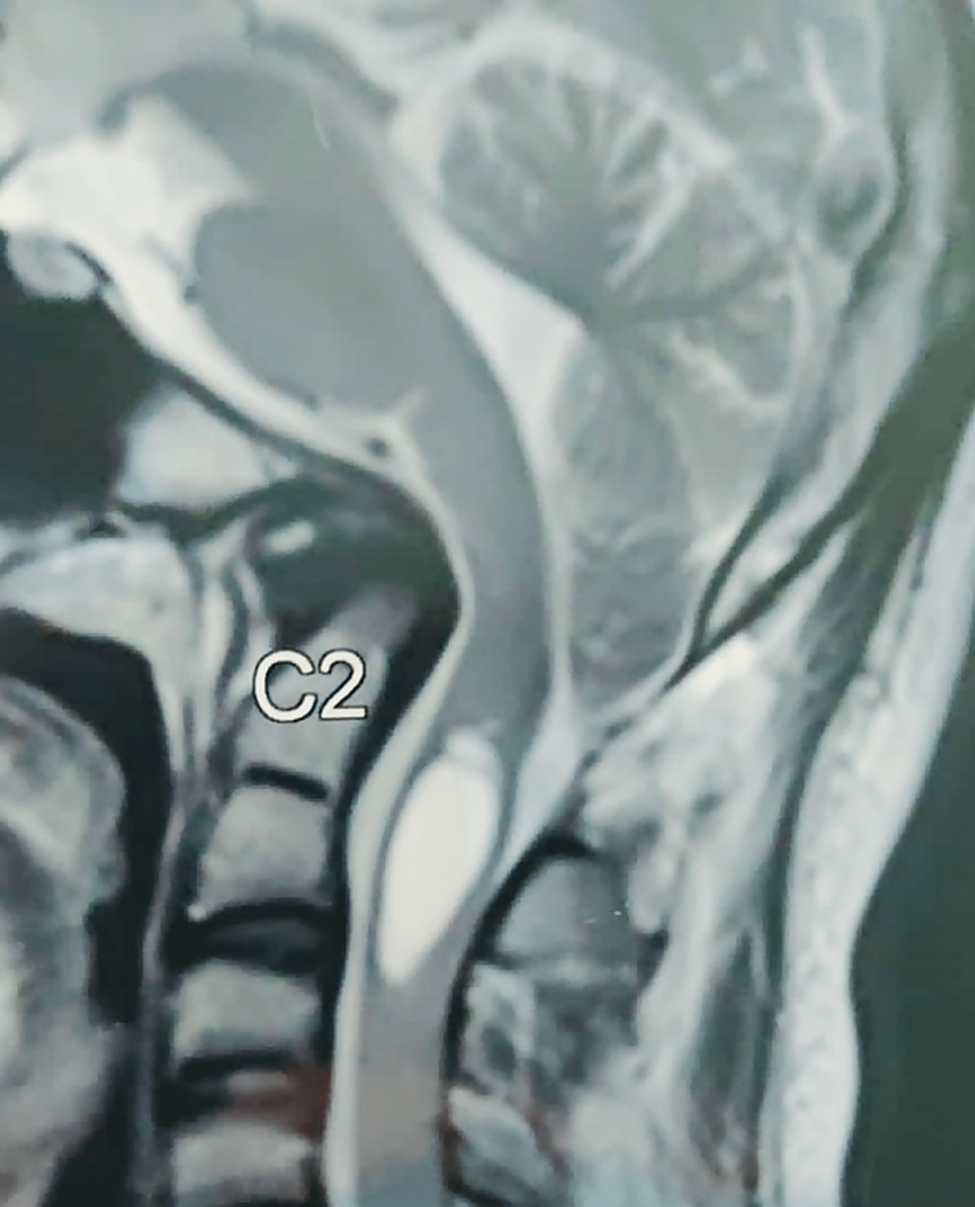
T2-weighted MRI (sagittal) showing cerebellar tonsil herniation through the foramen magnum with an intramedullary syrinx

A 16-year-old male presented with occipital headaches and lower neck pain for six months. Magnetic resonance imaging (MRI) revealed cerebellar tonsil herniation through the foramen magnum with an intramedullary syrinx, indicating ACM. (Figure 1)

**Figure 2:**
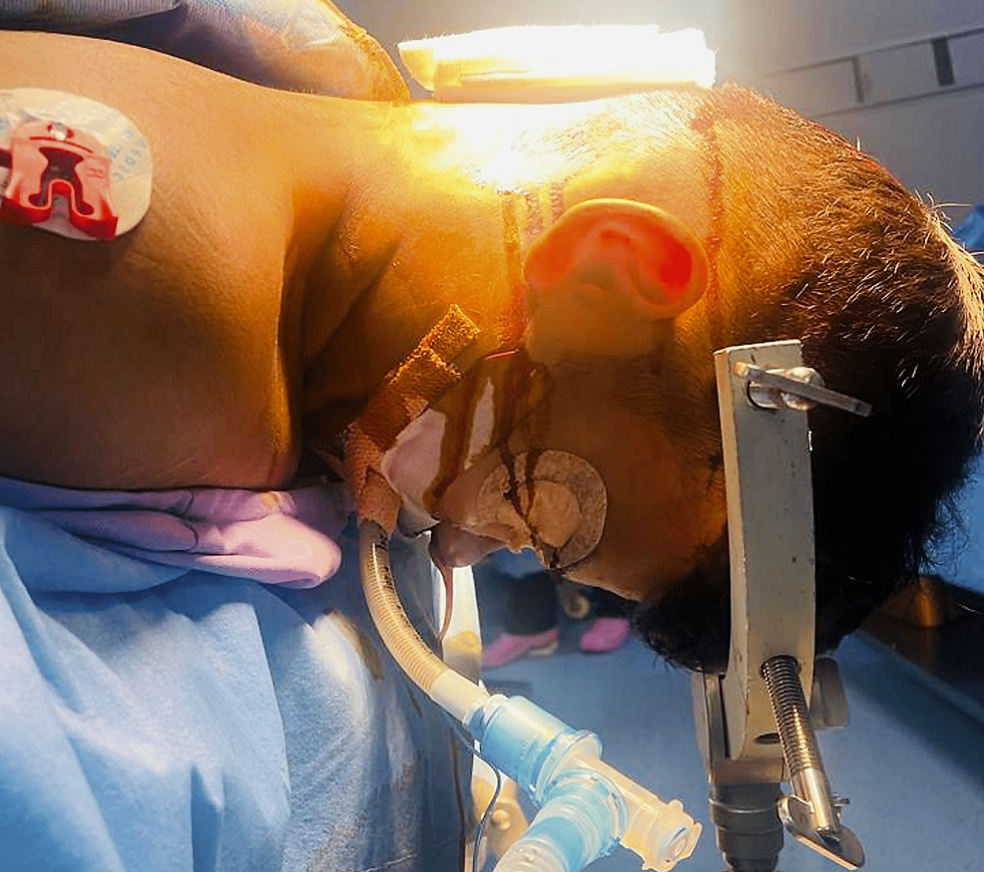
Patient in the prone position with the head fixed on the pin holder with extreme neck flexion for foramen magnum decompression

The patient was counselled and scheduled for foramen magnum decompression under general anaesthesia in the prone position. Pre-operative airway examination was unremarkable except for a mild restriction of head extension due to pain. After anaesthesia induction, a 7.0 ID reinforced tube was used for endotracheal intubation in the supine position, and a soft oral bite block (a roller gauge bite block) was placed in the midline without tongue compression. Anticipating bradyarrhythmia in these patient populations, a radial arterial line was secured for beat-to-beat cardiac monitoring. The patient was positioned prone, with the head held in place by a pin holder. The head and neck were fixed in full flexion as required for the suboccipital craniectomy, with the chin touching the sternum (Figure 2).

**Figure 3:**
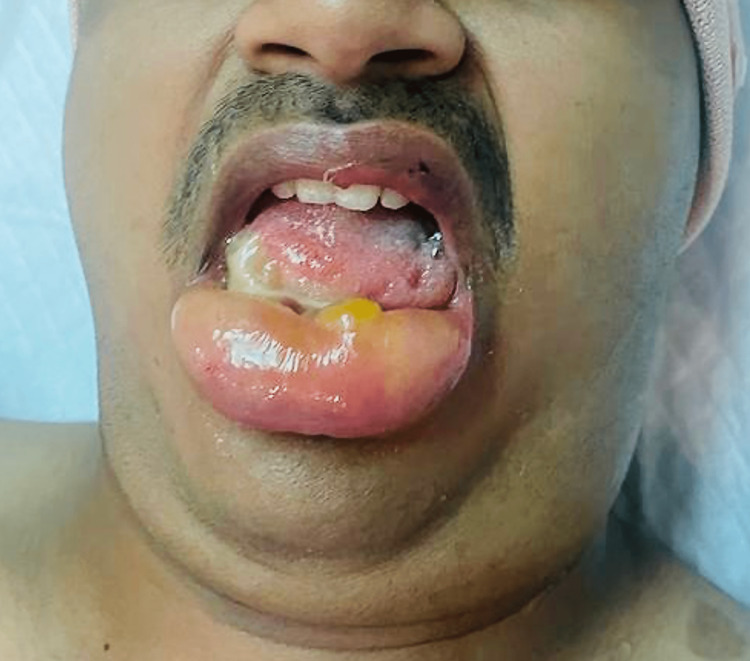
Facial and submandibular oedema with tongue and lip swelling in the immediate postoperative period.

The entire body was kept in a neutral position by using bolster pillows to support the chest and pelvis without abdominal compression. The anaesthesia was maintained with the minimum alveolar concentration (MAC 1) of sevoflurane and an atracurium infusion at 20 mg/hr. Fluids were titrated intravenously to achieve a pulse pressure variation of 12. At the end of the surgery, 1500 ml of normal saline was infused, and a urine output of 1000 ml was recorded. Because the patient had stable hemodynamics and acceptable blood loss, no blood products were given. The intraoperative surgical course was uneventful, lasting four hours, and a successful foramen magnum decompression with C1 arch excision was performed. The pin holder was removed after the procedure was completed, the patient was turned supine, and the oral bite block was removed. Clinically, there was significant swelling of the face, lower lip, and tongue, as well as a tense submandibular space (Figure 3).

**Figure 4:**
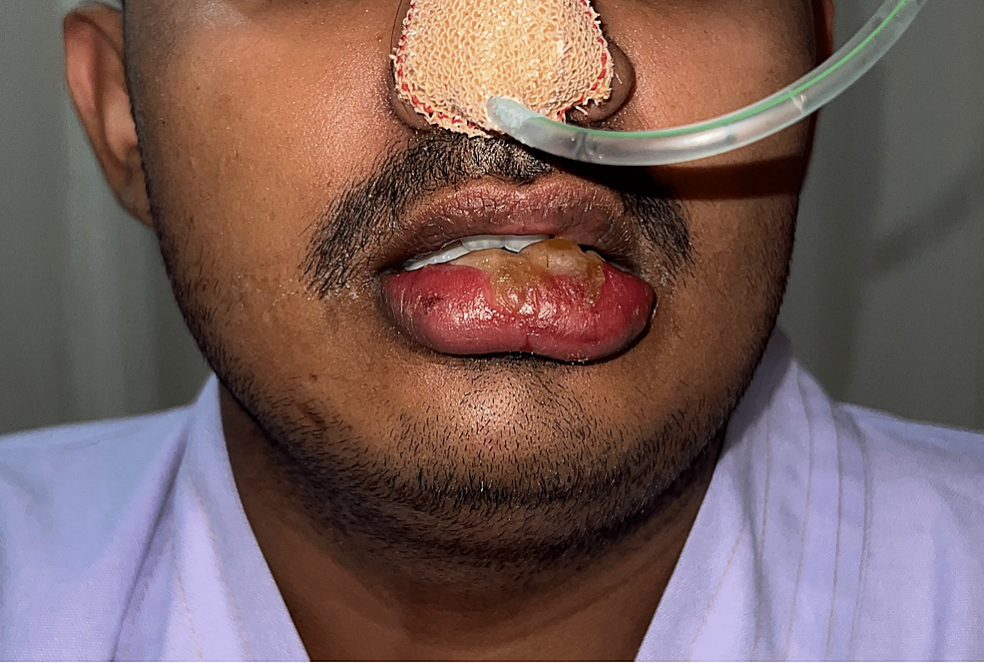
Resolution of oedema on the fifth postoperative day.

A pressure ulcer with a bleb was also noted over the lower lip. The possibility of an anaphylactic reaction was not considered because the patient was hemodynamically stable and had no skin rashes. The possibility of fluid overload was also ruled out as there was no conjunctival oedema and the chest was clear on auscultation. The oral cavity examination revealed oedema and congestion of the soft palate, uvula, and tonsillar pillars. Because of the prolonged intraoperative neck flexion, a provisional diagnosis of venous congestive oedema was considered. The patient was electively ventilated in the intensive care unit (ICU), and extubation was performed successfully after four hours because the patient was fully conscious. Prior to extubation, a cuff leak test was performed to rule out tracheal and laryngeal oedema. The patient was started on intravenous dexamethasone (24 mg/day) and was nursed in a head-up position. Close monitoring was performed in anticipation of upper airway obstruction and oxygen desaturation. On the advice of the otolaryngologist, nasogastric feeding was initiated on the first postoperative day. The patient was treated with serratiopeptidase, antibiotics (in view of the lip ulcer), and topical ice pack application. The patient was discharged from the hospital on the fifth postoperative day after there was a significant reduction in congestive swelling (Figure 4).

## Discussion

Prolonged surgical duration, head-down position, and excessive intravenous fluid infusion have all been shown to aggravate facial oedema in the prone position [2,3]. Extreme neck flexion during suboccipital craniectomy can also cause venous congestive oedema, which can be fatal [4]. Internal and external jugular vein compression due to neck flexion can impair venous drainage from the pharyngeal, lingual, and facial veins, resulting in facial and oropharyngeal swelling [5]. Furthermore, in a state of extreme neck flexion, the oral bite block can migrate intraorally, causing mechanical compression of the lingual arteries and exacerbating tongue swelling [6]. The postoperative swelling of the submandibular gland due to extreme neck flexion after retro-mastoid suboccipital craniotomies and other prone surgeries has been reported. [7,8]. When the neck is fully flexed, kinking and stretching of the salivary ducts, blood vessels, and lymphatics can cause obstruction and swelling of local structures. A tense submandibular swelling was also observed in our case, which can lead to immediate airway compromise following tracheal extubation.

Facial disfigurement caused by facial oedema can cause cosmetic dissatisfaction for the patient in the immediate postoperative period. Because a facial examination is not possible during suboccipital craniectomies under drapes, these complications may go undetected during the procedure. From the anesthesiologist’s perspective, maintaining a two-finger breadth space between the chin and the sternum after the prone position can effectively mitigate this complication caused by extreme neck flexion. We gave the patient extreme neck flexion, considering the kink-resistant nature of the flexo-metallic tube and to facilitate surgical exposure as required by the surgeon. Because oral bite blocks are required after the placement of reinforced endotracheal tubes in suboccipital craniectomies, the size of the roller gauge bite blocks must be customized, as longer bite blocks are more prone to compromise the blood supply of posterior pharyngeal structures in a state of extreme neck flexion. Prior to turning the patient prone, it is also critical to rule out lip entrapment between the incisors and bite block. Aside from the face, the entire body should be kept neutral with a free abdomen and pressure points protected with soft pads prior to surgical draping. To avoid an adverse respiratory event after tracheal extubation, it is critical to postpone extubation in the immediate postoperative period and closely monitor the airway in the ICU. Prior to extubation, a cuff leak test must be performed to rule out laryngeal or tracheal oedema in the presence of lower neck swelling. Immediate postoperative oral feeding should be avoided until oropharyngeal oedema resolves, and nasogastric feeding can help with patient nutrition. Intravenous steroids are critical in reducing inflammation and oedema, and antibiotics should be supplemented to prevent venous or pressure mucosal ulcer infection. Intravenous analgesics are required to alleviate pain caused by oropharyngeal structure congestion.

## Conclusions

From a surgical standpoint, good neck flexion is required for a successful foramen magnum decompression with C1 arch excision for ACM. Endotracheal intubation with a reinforced tube can help prevent tube kinking and ensuring a two-finger breadth space between the chin and the sternum before surgery is critical to prevent life-threatening congestive oedema.

## References

[1] Rao D, Le RT, Fiester P, Patel J, Rahmathulla G (2020). An illustrative review of common modern craniotomies. J Clin Imaging Sci.

[2] Kwee MM, Ho YH, Rozen WM (2015). The prone position during surgery and its complications: a systematic review and evidence-based guidelines. Int Surg.

[3] Koreckij J, Price N, Schwend RM (2011). Vectored cranial-cervical traction limits facial contact pressure from prone positioning during posterior spinal deformity surgery. Spine (Phila Pa 1976).

[4] Hsu S, Hsieh C, Huang C, Huang J (2012). Delayed airway obstruction in posterior fossa craniotomy with park-bench position—a case report and review of the literatures. Surg Sci.

[5] Sinha A, Agarwal A, Gaur A, Pandey CK (2001). Oropharyngeal swelling and macroglossia after cervical spine surgery in the prone position. J Neurosurg Anesthesiol.

[6] Koizumi H, Utsuki S, Inukai M, Oka H, Osawa S, Fujii K (2012). An operation in the park bench position complicated by massive tongue swelling. Case Rep Neurol Med.

[7] Nakanishi H, Tono T, Ibusuki S (2015). Postoperative submandibular gland swelling following craniotomy under general anesthesia. Case Rep Otolaryngol.

[8] Hans P, Demoitié J, Collignon L, Bex V, Bonhomme V (2006). Acute bilateral submandibular swelling following surgery in prone position. Eur J Anaesthesiol.

